# Long‐term music adjuvant therapy enhances the efficacy of sub‐dose antiepileptic drugs in temporal lobe epilepsy

**DOI:** 10.1111/cns.13623

**Published:** 2021-02-28

**Authors:** Ceng‐Lin Xu, Jia‐Zhen Nao, Yu‐Jia Shen, Yi‐Wei Gong, Bei Tan, Shuo Zhang, Ke‐Xin Shen, Cui‐Rong Sun, Yi Wang, Zhong Chen

**Affiliations:** ^1^ Institute of Pharmacology & Toxicology College of Pharmaceutical Sciences Zhejiang University Hangzhou China; ^2^ Key Laboratory of Neuropharmacology and Translational Medicine of Zhejiang Province Zhejiang Chinese Medical University Hangzhou China; ^3^ Institute of Drug Metabolism and Analysis College of Pharmaceutical Sciences Zhejiang University Hangzhou China

**Keywords:** Mozart K.448, music adjunctive therapy, sub‐dose antiepileptic drugs, temporal lobe epilepsy

## Abstract

**Aims:**

Noninvasive music adjuvant therapy shows great potential in improving seizure control when combined with routine antiepileptic drugs. However, the diversity of previous music protocols has resulted in disparate outcomes. The optimized protocol and features for music adjuvant therapy are still not fully understood which limits its feasibility.

**Methods:**

By applying different regimens of music therapy in various temporal lobe epilepsy models, we evaluated the effect of music in combination with sub‐dose drugs on epileptic seizures to determine the optimized protocol.

**Results:**

A subgroup of kindled mice that were responsive to music adjuvant therapy was screened. In those mice, sub‐dose drugs which were noneffective on kindled seizures, alleviated seizure severity after 12 h/day Mozart K.448 for 14 days. Shorter durations of music therapy (2 and 6 h/day) were ineffective. Furthermore, only full‐length Mozart K.448, not its episodes or other music varieties, was capable of enhancing the efficacy of sub‐dose drugs. This music therapeutic effect was not due to increasing cerebral drug concentration, but instead was related with the modulation of seizure electroencephalogram (EEG) spectral powers in the hippocampus.

**Conclusion:**

These results indicate that long‐term full‐length Mozart K.448 could enhance the anti‐seizure efficacy of sub‐dose drugs and may be a promising noninvasive adjuvant therapy for temporal lobe epilepsy.

## Graphical abstract

Music adjuvant therapy promotes the efficacy of sub‐dose antiepileptic drugs in both hippocampal and amygdaloid kindled temporal lobe epileptic mice. Listening to full‐length Mozart K.448 for 12 h/day is the optimal protocol for music adjuvant therapy.

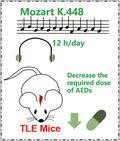

## 1 INTRODUCTION

Epilepsy is one of the world's most common neurological disease and is characterized by recurrent abrupt seizures, which burden or even threaten the lives of patients.^1^ Antiepileptic drugs (AED) are the preferred choice to control seizures^2^, ^3^; however, the high incidence of pharmacoresistance, narrow therapeutic index, and side‐effects caused by long‐term pharmacotherapeutics raises substantial concerns.^4^, ^5^ Other approaches, such as alternative epileptic surgery or deep brain stimulation, are not suitable for all the patients and have the potential to cause irreversible lesions.^6^, ^7^ Recently, the development of noninvasive adjuvant therapies—like music therapy—has created a new path for helping to control seizures in epilepsy. Whether these adjuvant therapies can enhance the efficacy of AEDs is an on‐going critical issue in this field.^8^

Music is an essential part of daily life and has been reported to affect various neurological functions, such as mood, emotion, memory, and other mental states.^9^, ^10^ In the recent decade, increasing evidence has highlighted the adjuvant therapeutic role of music‐based interventions in many chronic brain disorders such as Parkinson disease, Alzheimer disease, and epilepsy.^11^, ^12^ In particular, clinical evidence has demonstrated the beneficial role of Mozart's sonata K.448 in helping reducing epileptic spikes and controlling seizures in genetic epilepsy.^13^, ^14^ However, several major questions remain unanswered about the efficacy of music therapy. First, previous studies have primarily focused on epilepsy which is caused by cortical originated abnormal discharges. However, whether music has an analogous favorable effect on temporal lobe epilepsy (TLE)—which is the most common type of pharmacoresistant epilepsy—remains unclear. Second, the majority of patients are still taking routine AEDs while exposed to music. As such their interaction, specifically whether music can reduce the needed dose of AEDs, warrants further investigation. Last and most importantly, a significant diversity with respect to therapeutic protocols of music adjuvant therapy—including type of music, duration of listening to music, etc.—is prevalent in the studies. These discrepancies may be the root causes leading to the varied effects of Mozart K.448 among studies.^14^, ^15^, ^16^, ^17^ Considering the complicated mechanisms of music therapy, an optimized protocol for music therapy is urgently needed.

In the present study, by applying multiple protocols of music adjuvant therapy on different TLE models, we tested the effect of music combined with sub‐dose AEDs on TLE seizures and determined the optimized protocols for music adjuvant therapy.

## 2 MATERIALS AND METHODS

### 2.1 Animals

Male C57BL mice (25–30 g, Grade II, Experimental Animal Center, Zhejiang Academy of Medical Science) were used in this study. The mice were kept in cages with a 12 h light/dark cycle (lights on from 8:00 to 20:00) and provided with *ad libitum* food and water. All experiments were carried out in accordance with the ethical guidelines of the Zhejiang University Animal Experimentation Committee and were in complete compliance with the ARRIVE guidelines for the Care and Use of Laboratory Animals. Sample sizes were chosen to reliably measure experimental parameters and are listed in the figure legends.

### 2.2 Surgery

To implant electrodes into the hippocampus or the amygdala, mice were anesthetized with sodium pentobarbital (50 mg/kg) and mounted on a stereotaxic apparatus (RWD Life Science). Bipolar electrodes, made of stainless‐steel Teflon‐coated wires (791500, A.M. Systems; diameter 0.125 mm; distance between exposed tips 0.5 mm), were implanted into the right ventral CA3 (AP: −2.9 mm; ML: −3.2 mm; DV: −3.2 mm) or the right basal lateral amygdala (BLA, AP: −1.2 mm; ML: −2.6 mm; DV: −4.9 mm). The coordinates were measured from the bregma according to the mouse atlas.^18^ The reference and ground screws were placed in the bone over the cerebellum. Then, the recording and reference electrodes were welded to a receptacle. Mice were kept for one‐week post‐surgery to recover.

### 2.3 Rapid hippocampal kindling epilepsy model

Rapid hippocampal kindling was performed following the precise procedure from our previous studies.^19^, ^20^, ^21^ Briefly, the after‐discharge threshold (ADT) of each mouse was determined (monophasic square‐wave pulses, 20 Hz, 1 ms/pulse, 40 pulses) with a constant‐current stimulator (SEN‐7203, SS‐202 J; Nihon kohden) and electroencephalograms (EEGs) were recorded with a Neuroscan system (NuAmps, Neuroscan System). The initial intensity started at 40 μA and was then increased in 20 μA steps with an 1 min interval. The minimal intensity that evoked at least a 5 s after‐discharge duration (ADD) was defined as the ADT. On the next day, kindling stimulations were carried out on each mouse. All mice received 10 kindling stimulations daily with a current density of 400 μA (20 Hz, 2 s trains, 1 ms monophasic square‐wave pulses) at an interval of 30 min. The behavioral stage was recorded according to the Racine's classification (1, mouth and facial movement; 2, head nodding; 3, forelimb clonus; 4, rearing with forelimb clonus; and 5, rearing and falling with forelimb clonus).^22^ Mice that experienced three consecutive stage five seizures were considered as fully kindled.

### 2.4 Amygdala kindling epilepsy model

ADTs were determined in each mouse by the constant‐current stimulator (monophasic square‐wave pulses, 60 Hz, 1 ms/pulse, 60 pulses). The initial intensity was started at 60 μA and was then increased in 20 μA steps every 10 min. The minimal intensity to evoke >5 s ADD was determined as the ADT. From the next day on, mice were kindled by ADT twice a day, with an interval of 4 h. The observation of behavioral stages and estimation of full kindled were identical to the protocol used in the rapid hippocampal kindling.

### 2.5 Music and antiepileptic drugs treatments

Fully kindled mice were treated with Mozart K.448^23^ or other music during the active state (20:00–8:00). Mice were kept in a soundproof box (Jiliang Software Technology) 30 min before applying Mozart K.448 for adaption. Mozart K.448 was applied through a loudspeaker placed 70–100 cm above the mice. The sound volume was 75 dB.^24^ Effects of valproic acid (VPA, 50,100 and 200 mg/kg, Sigma Aldrich) or levetiracetam (LEV, 1 and 3 mg/kg, Sigma Aldrich) on kindled seizures were determined before Mozart K.448 application, and 7 and 14 days after. Kindled ADTs, which were used for the kindled seizure stimulation of each mouse, were determined before each VPA test with the same current parameters as the initial ADTs. One hour later, mice before receiving AEDs injection were firstly kindled with the ADTs, and the seizure severity of kindled mice including the seizure stage, the ADD, and the generalized seizure duration (GSD) was recorded as self‐control (the pre group). Then, VPA or LEV was injected intraperitoneally 1 h later. 30 min after injection, mice were kindled and the seizure severity was recorded. Given that in kindling models, stages 1–3 were considered to be focal seizures and stages 4–5 were considered as severer generalized seizures,^25^ once the seizure stage of the mice was inhibited to stage 3 or less after receiving AEDs, these mice were considered responsive and are hereafter referred to the response group (RES). The other mice, which still exhibited stage 4 or 5 seizures, were considered non‐responsive and referred to the non‐response group (NON). The seizure stage, ADD, and GSD were scored by a researcher blinded to the group allocation.

### 2.6 EEG analysis

EEG recording and spectrum analysis were evaluated in a manner consistent with our previous reports.^26^, ^27^ Seizure EEG of CA3 was recorded with a Neuroscan system with band‐pass filters spanning from 0–200 Hz and sampling rate at 1000 Hz. The entire seizure EEG were selected manually and analyzed offline by Scan 4.5. Each seizure EEG was digitally band‐pass filtered from 0.3 to 100 Hz and then divided into consecutive 4 s epochs (4096 points) on which fast Fourier transform (FFT) was run with a Hanning window to avoid edge effects. The FFT output provided a total power for each 4 s epoch. These frequency bins were subsequently averaged within 5 frequency bands: delta, 0.5–4 Hz; theta, 4–8 Hz; alpha, 8–12 Hz; beta, 12–30 Hz; gamma, 30–100 Hz.^21^

The sum of the distances between consecutive data points (coastline index) of each seizure EEG was calculated according to our previous studies.^27^, ^28^ An algorithm was used to calculate the sum of the absolute value of the distances from one data point to the next. The calculation was performed on MATLAB 9.0 (Mathworks).

### 2.7 Determination of intracerebral drug concentrations

To compare the intracerebral VPA concentrations between the RES and NON groups, micro‐dialysis was performed following the protocol of our previous study.^29^ Micro‐dialysis probes (MAB) were implanted into the right dorsal hippocampal CA3 (AP: −2.8 mm; ML: −3.2 mm; DV: −0.7 mm) of mice from both groups through the guide cannula. Micro‐dialysis was then initiated by connecting the probe inlet to a microinjection pump system (CMA) that circulated the probe continuously with artificial cerebrospinal fluid (ACSF, 125 mM NaCl, 2.5 mM KCl, 1.26 mM CaCl_2_, 1.18 mM MgCl_2_) at a rate of 1.5 μL/min. *In vitro* recovery value was determined before microdialysis probes were used for *in vivo* experiments, to calculate the corrected dialysate concentrations. For *in vivo* experiments, the perfusion exudate from the first 90 min was discarded. Then, mice were i.p. injected with VPA (100 mg/kg) and samples were collected at 30, 60, 90 and 120 min thereafter (the sample size was 45 μL). All micro‐dialysis samples were immediately frozen at −80°C and stored until analysis by high‐performance liquid chromatography‐mass spectrometry (HPLC‐MS/MS, Shimadzu). For VPA concentration determination, 20 μL dialysate of each sample was pretreated by mixture with 80 μL 10% methanol. The VPA concentration of dialysate was then measured via HPLC‐MS. Chromatographic conditions were as follows: C18 column (100 × 2.1 mm, 5 μm, Thermo), 10 μL injection volume; mobile phase, acetonitrile/water (with 10 mmol/L ammonium acetate; 5:95, v:v); flow rate, 0.45 mL/min; column temperature, 40℃; sample disk temperature, 4℃. Mass spectrometry parameters were as follows: ESI source, negative ion mode; SIM mode, m/z 143.0; dwell time, 100 ms. Interface parameters: nebulizer gas flow, 3 L/min; heating gas flow, 10 L/min; interface temperature, 300℃; DL temperature, 250℃; heating block temperature, 400℃; drying gas flow, 10 L/min.

### 2.8 Statistics analysis

All data are presented as means ± S.E.M. Statistical comparisons were performed with GraphPad prism (version 6.0). Whether the data followed a Gaussian distribution was tested first. If yes, unpaired or paired *t*‐tests were used for two groups comparison. If not, nonparametric Wilcoxon or Mann‐Whitney tests were used instead. The detailed statistics are indicated in the figure legends. A *p* < 0.05 was considered to be a significant difference.

## 3 RESULTS

### 3.1 Long‐term Mozart K.448 enhances the anti‐seizure efficacy of sub‐dose VPA on hippocampal kindled seizures

**FIGURE 1:**
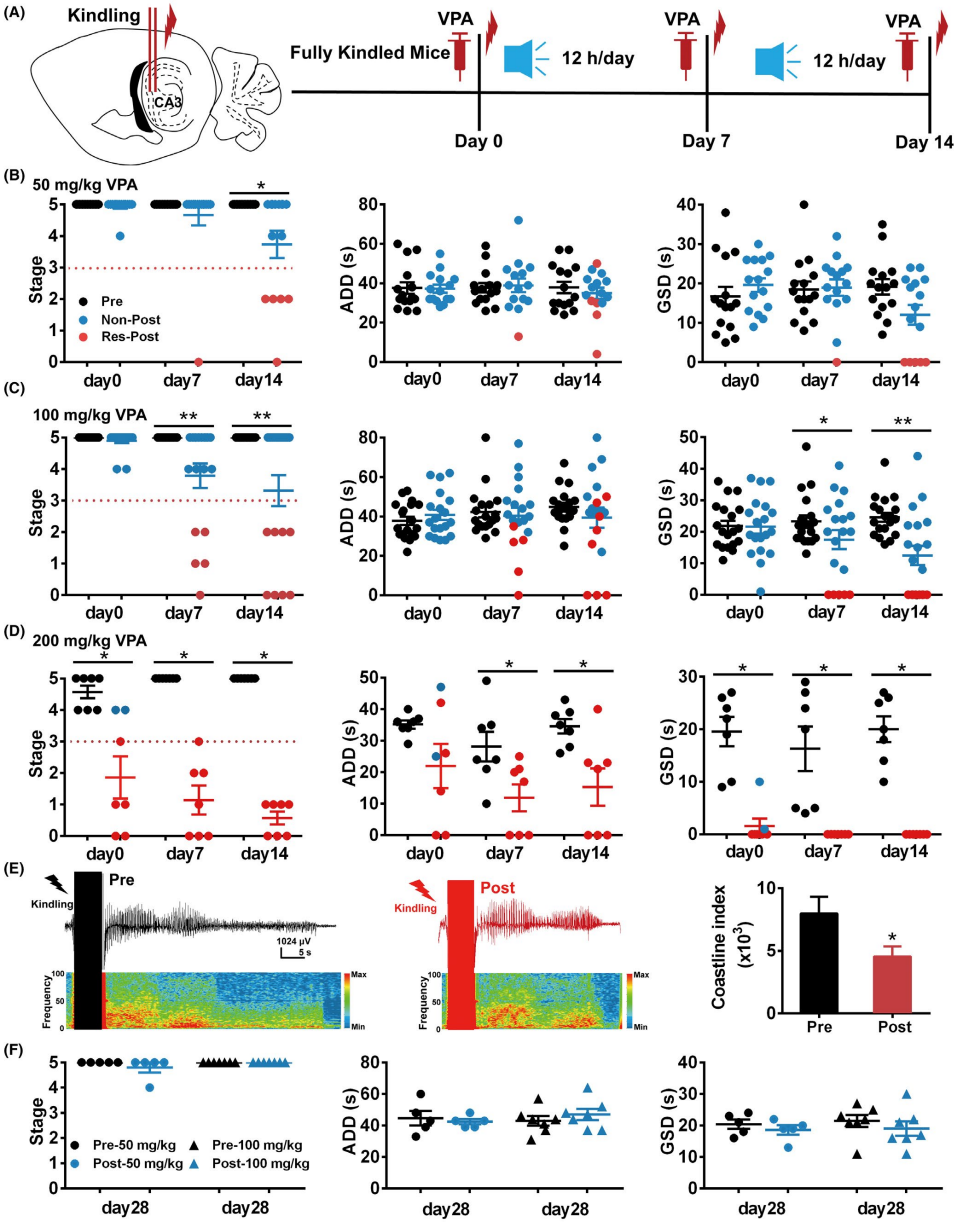
Application of 12 h/day Mozart K.448 for 14 days enhances the anti‐seizure efficacy of VPA on hippocampal kindled mice. (A) Schematic diagram of the experimental design for application of 12 h/day Mozart K.448. (B) Mozart K.448 therapy enhances the anti‐seizure efficacy of 50 mg/kg VPA on kindled seizures (n = 15; for seizure stage on day 14, ^*^ *p* = 0.016, nonparametric Wilcoxon test was used). (C) Mozart K.448 therapy enhances the anti‐seizure efficacy of 100 mg/kg VPA on kindled seizures (n = 19; for seizure stage on day 7, ^**^ *p* = 0.0039; for seizure stage on day 14, ^**^ *p* = 0.0078; for GSD on day 7, ^*^ *p* = 0.020, t = 2.57, df =18; for GSD on day 7, ^*^ *p* = 0.037; for GSD on day 14, ^**^ *p* = 0.0035, nonparametric Wilcoxon test was used). (D) Mozart K.448 therapy enhances the anti‐seizure efficacy of 200 mg/kg VPA on kindled seizures (n = 7; for seizure stage on day 0, ^*^ *p* = 0.031; for seizure stage on day 7 and day 14, ^*^ *p* = 0.016; nonparametric Wilcoxon test was used; for ADD on day 7, ^*^ *p* = 0.016; nonparametric Wilcoxon test was used; for ADD on day 14, ^*^ *p* = 0.021; paired t‐test was used; for GSD on day 0, day 7 and day 14, ^*^ *p* = 0.016, nonparametric Wilcoxon test was used). (E) Left and middle panel: representative seizure EEGs before and after 100 mg/kg VPA in one mouse from the RES group after Mozart K.448 therapy. Right plot: statistical analysis of coastline index of seizure EEGs before and after VPA in mice from the RES group (n = 8, ^*^ *p* = 0.023, nonparametric Wilcoxon test was used). (F) Extending Mozart K.448 application still has no effect on promoting the effect of both 50 and 100 mg/kg VPA in mice from the NON group (n = 5, for 50 mg/kg VPA; n = 7 for 100 mg/kg VPA)

First, we aimed to test the effects of Mozart K.448 in combination with VPA (50, 100 and 200 mg/kg) on hippocampal kindled seizures (Figure 1A). The drug effect on kindled seizures was tested at 0 (before), 7, and 14 days after listening to Mozart K.448 (12 h/day). At day 0, both 50, and 100 mg/kg VPA showed no anti‐seizure effect on kindled mice. However, by day 7 an anti‐seizure impact emerged for both doses, becoming more prominent by day 14. At day 14, both 50 and 100 mg/kg VPA reduced the seizure stage and GSDs on a subgroup (approximately 40%) of kindled mice (5 out of 15 mice for 50 mg/kg; 8 out of 19 mice for 100 mg/kg), these mice were designated as the RES group, while the other mice were classified as the NON group (Figure 1B and C). Unlike sub‐dose VPA, 200 mg/kg VPA (therapeutic dose) inhibited seizures at day 0, and this anti‐seizure effect could be further enhanced by listening to Mozart K.448 at day 7 and day 14 for all 7 mice (Figure 1D). It can be observed that the anti‐seizure effect of music adjuvant therapy plus sub‐dose VPA is similar to therapeutic dose VPA in the RES group (Figure 1B‐D). Further EEG analysis showed that after listening to Mozart K.448, sub‐doses of drugs could also reduce the coastline index of seizure EEGs in the RES group (Figure 1E). We then extended the music application to 4 weeks in the NON group to see whether longer music would be effective. Even so, neither 50 nor 100 mg/kg VPA could not inhibit seizures at day 28 in the NON group (Figure 1F), suggesting the non‐response to music seems to be permanent in that group.

**FIGURE 2:**
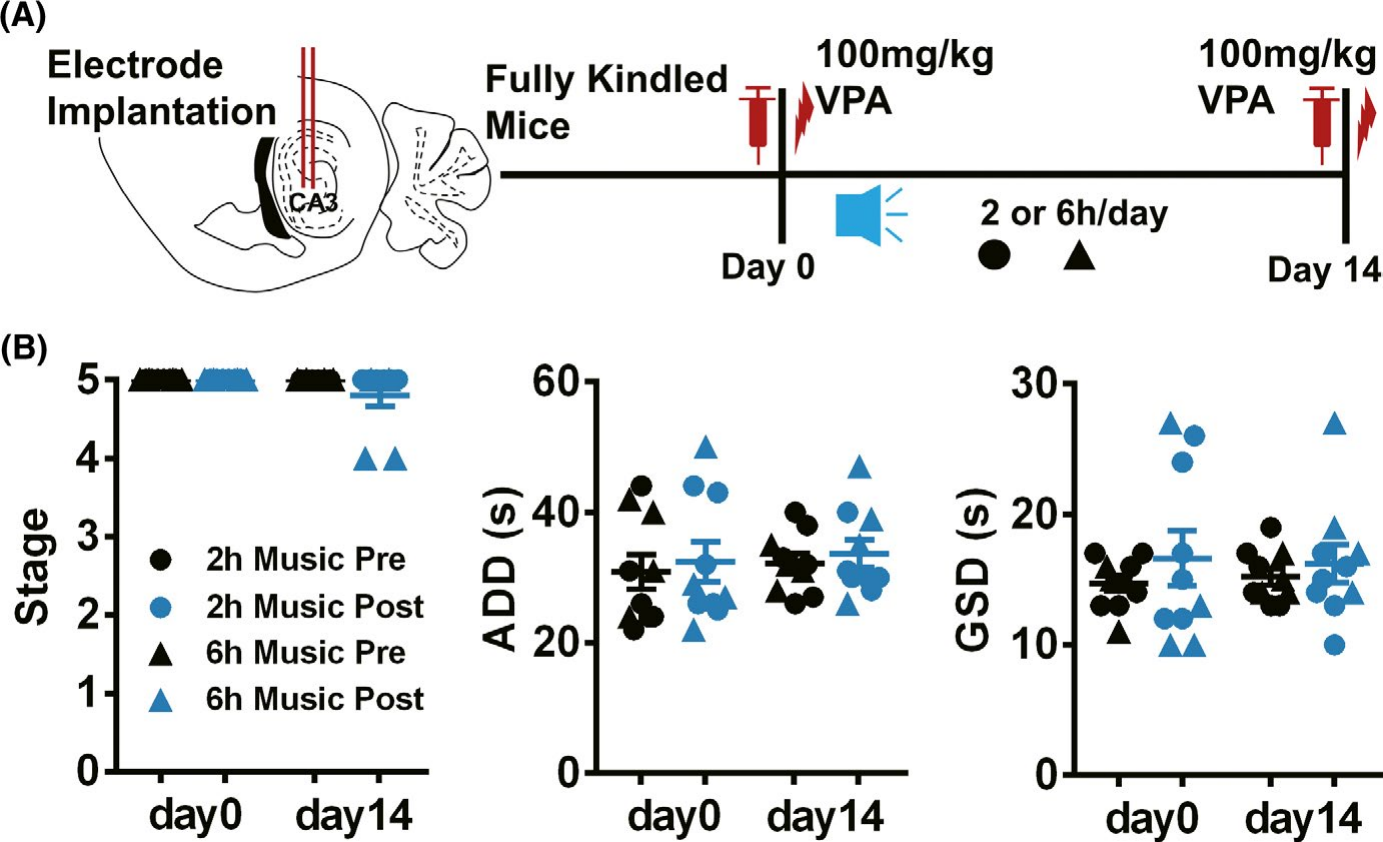
Application of 2 and 6 h/day Mozart K.448 for 14 days is ineffective in enhancing the anti‐seizure efficacy of VPA on hippocampal kindled mice. (A) The schematic diagram of the experimental design for application of 2 or 6 h/day Mozart K.448. (B) Both 2 and 6 h/day Mozart K.448 could not enhance the efficacy of VPA on hippocampal kindled mice (n = 5 for 2 h/day, marked with filled circles; n = 4 for 6 h/day, marked with filled triangles, neither short‐term music protocols plus VPA could decrease the seizure stage to less than 3, indicating the responsive rate is <20%)

Given that the music listening duration is a crucial parameter to be determined, we further tested whether a shortened time of listening to Mozart K.448 would have similar effect as the 12 h/day protocol. Thus, we applied 2 or 6 h/day Mozart K.448 on hippocampal kindled mice, respectively (Figure 2A).^16^, ^30^ By day 14 of 100 mg/kg VPA treatment, neither listening to 2 nor 6 h/day Mozart K.448 induced an anti‐seizure effect on kindled mice (Figure 2B, usually 4–5 mice were tested at first, if none of these mice was response to 100 mg/kg VPA, which indicating the responsive rate is <20%, this protocol was considered noneffective and no further experiments were performed to extend the sample size). In totality, the above results indicate that 12 h/day Mozart K.448 could enhance the anti‐seizure efficacy of sub‐dose VPA on hippocampal kindled TLE seizures.

### 3.2 Mozart K.448 has the therapeutic potential extending to different kindled models and AEDs

**FIGURE 3:**
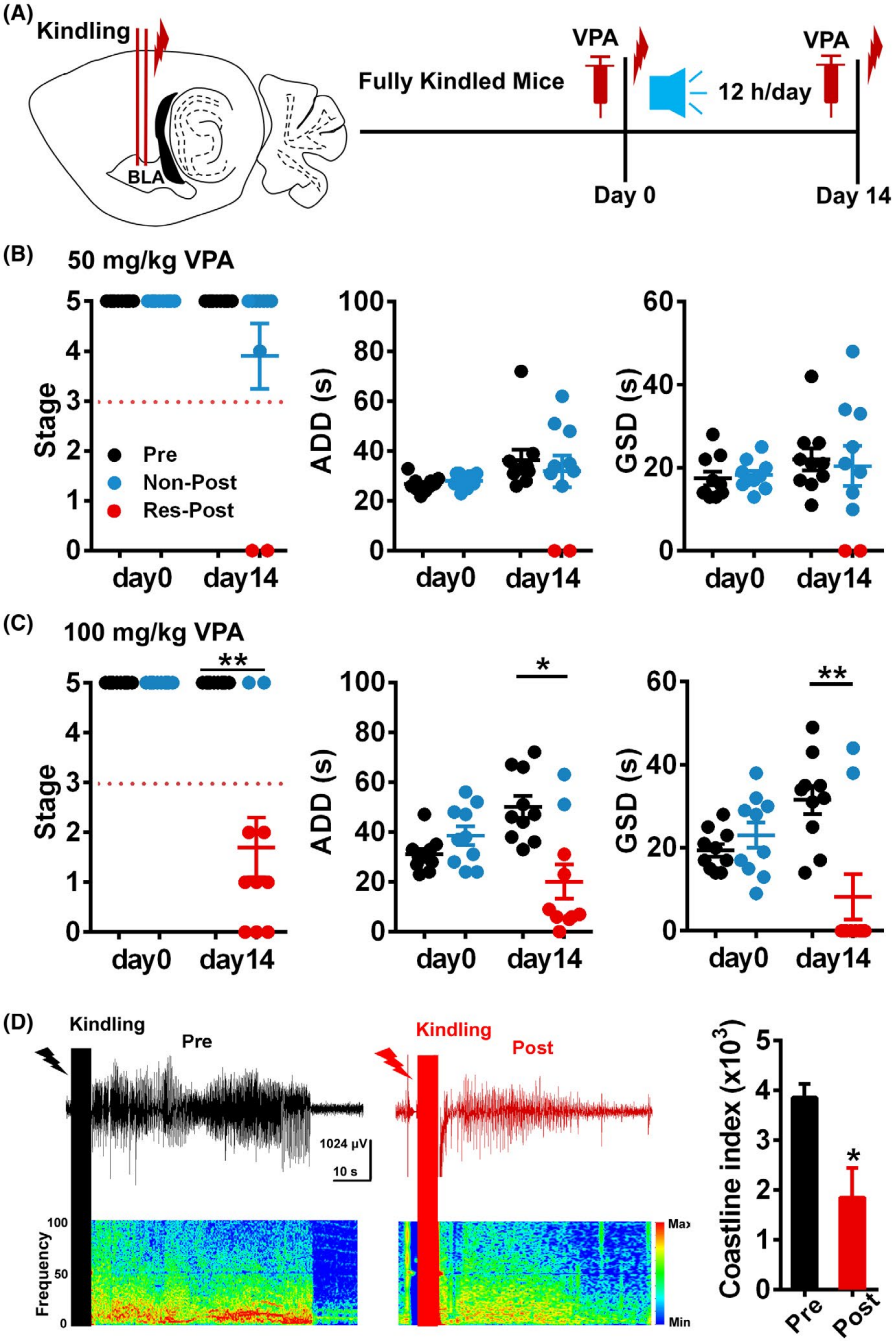
Mozart K.448 has the therapeutic potential extending to different kindled models. (A) The schematic diagram of the experimental design for application of Mozart K.448 on amygdala kindled mice. (B) Mozart K.448 therapy slightly enhances the anti‐seizure efficacy of 50 mg/kg VPA on amygdala kindled seizures (n = 10). (C) Mozart K.448 therapy enhances the anti‐seizure efficacy of 100 mg/kg VPA on kindled seizures (n = 10; for seizure stage on day 14, ^**^ *p* = 0.0078; for ADD on day 14, ^*^ *p* = 0.020; for GSD on day 14, ^**^ *p* = 0.0098, nonparametric Wilcoxon test was used). (D) Left and middle panel: representative seizure EEGs before and after 100 mg/kg VPA in one mouse from the RES group after Mozart K.448 therapy. Right plot: statistical analysis of coastline index of seizure EEGs before and after VPA in mice from the RES group (n = 6, ^*^ *p* = 0.031, nonparametric Wilcoxon test was used)

Considering that seizures in TLE patients often originate from different foci, we then further tested whether Mozart K.448 had similar effect on another kindled model whose seizure focus is amygdala (Figure 3A). As in the above experiment, both 50 and 100 mg/kg VPA had no anti‐seizure effect at day 0, but inhibited seizure stages, GSDs, and coastline index at day 14 (2 of 10 mice for 50 mg/kg; 8 of 10 mice for 100 mg/kg) for kindled mice (Figure 3B‐D). These results indicated that Mozart K.448 (12 h/day) can enhance the anti‐seizure efficacy of sub‐dose VPA on different kindled models.

**FIGURE 4:**
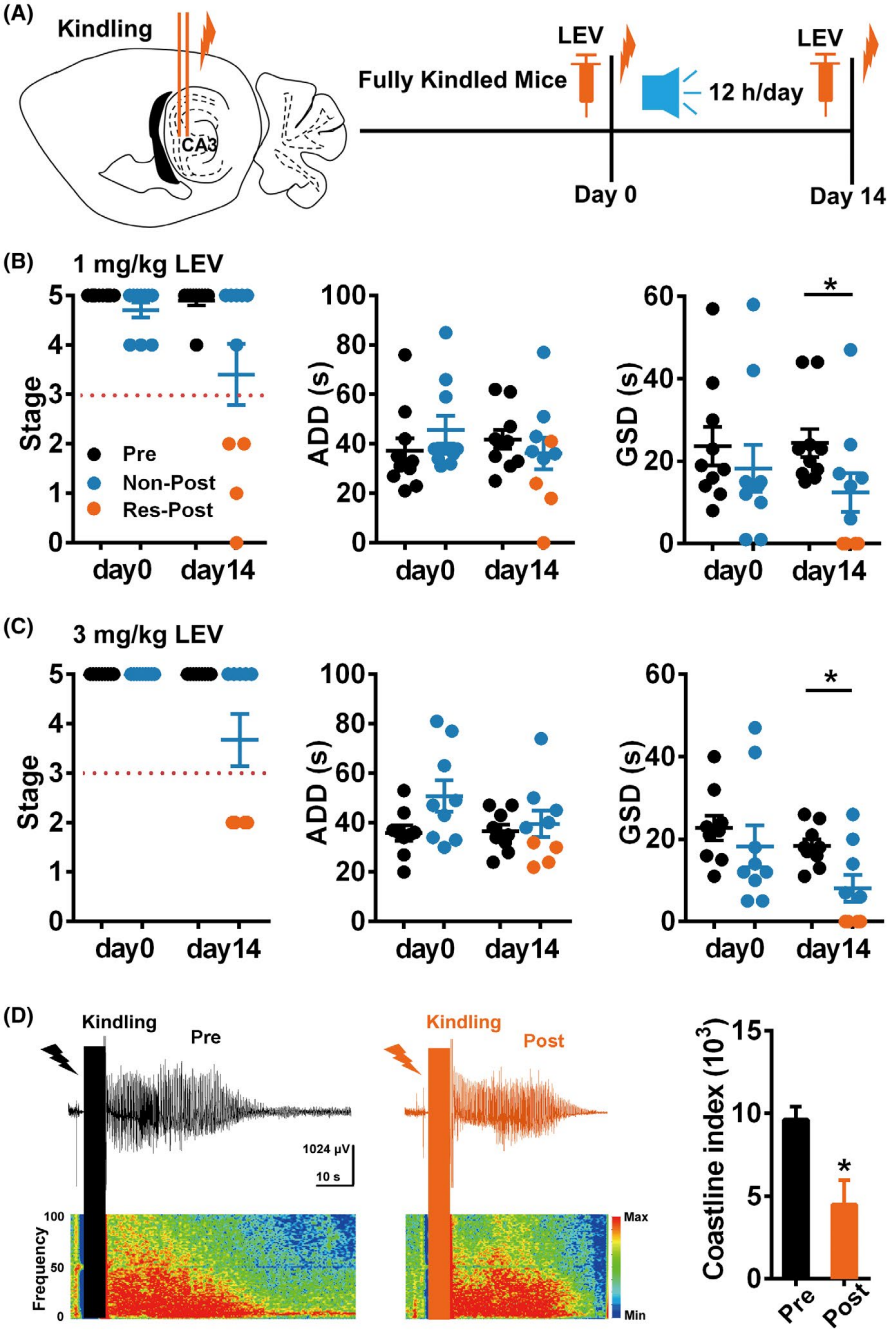
Mozart K.448 has the therapeutic potential extending to different AEDs. (A) The schematic diagram of the experiment design for testing the therapeutic effect of Mozart K.448 combined with LEV. (B) Mozart K.448 therapy enhances the anti‐seizure efficacy of 1 mg/kg LEV on kindled seizures (n = 10; for GSD on day 14, ^*^ *p* = 0.037, nonparametric Wilcoxon test was used). (C) Mozart K.448 therapy enhances the anti‐seizure efficacy of 3 mg/kg LEV on kindled seizures (n = 9; for GSD on day 14, ^*^ *p* = 0.020, nonparametric Wilcoxon test was used). (D) Left and middle panel: representative seizure EEGs before and after 3 mg/kg LEV in one mouse from the RES group after Mozart K.448 therapy. Right plot: statistical analysis of coastline index of seizure EEGs before and after LEV in mice from the RES group (n = 6, ^*^ *p* = 0.031, nonparametric Wilcoxon test was used)

As various AEDs with different mechanisms are chosen for TLE patients based on symptoms, we also tested the effect of Mozart K.448 combined with sub‐dose LEV (1 and 3 mg/kg) on hippocampal kindled seizures (Figure 4A). Sub‐dose 1 and 3 mg/kg LEV had no effect on kindled seizures at day 0, but inhibited seizure stage and GSDs (4 of 10 mice for 1 mg/kg; 2 of 7 mice for 3 mg/kg) after listening Mozart K.448 for 14 days (Figure 4B and C). The severity of seizure EEG was also reduced (Figure 4D). Taken together, these results indicated that Mozart K.448 (12 h/day) can enhance the efficacy of various AEDs.

In totality, the above results indicate that 12 h/day Mozart K.448 had the therapeutic potential extending to different kindled models and AEDs.

### 3.3 Full‐length Mozart K.448 is necessary for enhancing the anti‐seizure efficacy of VPA on hippocampal kindled mice

**FIGURE 5:**
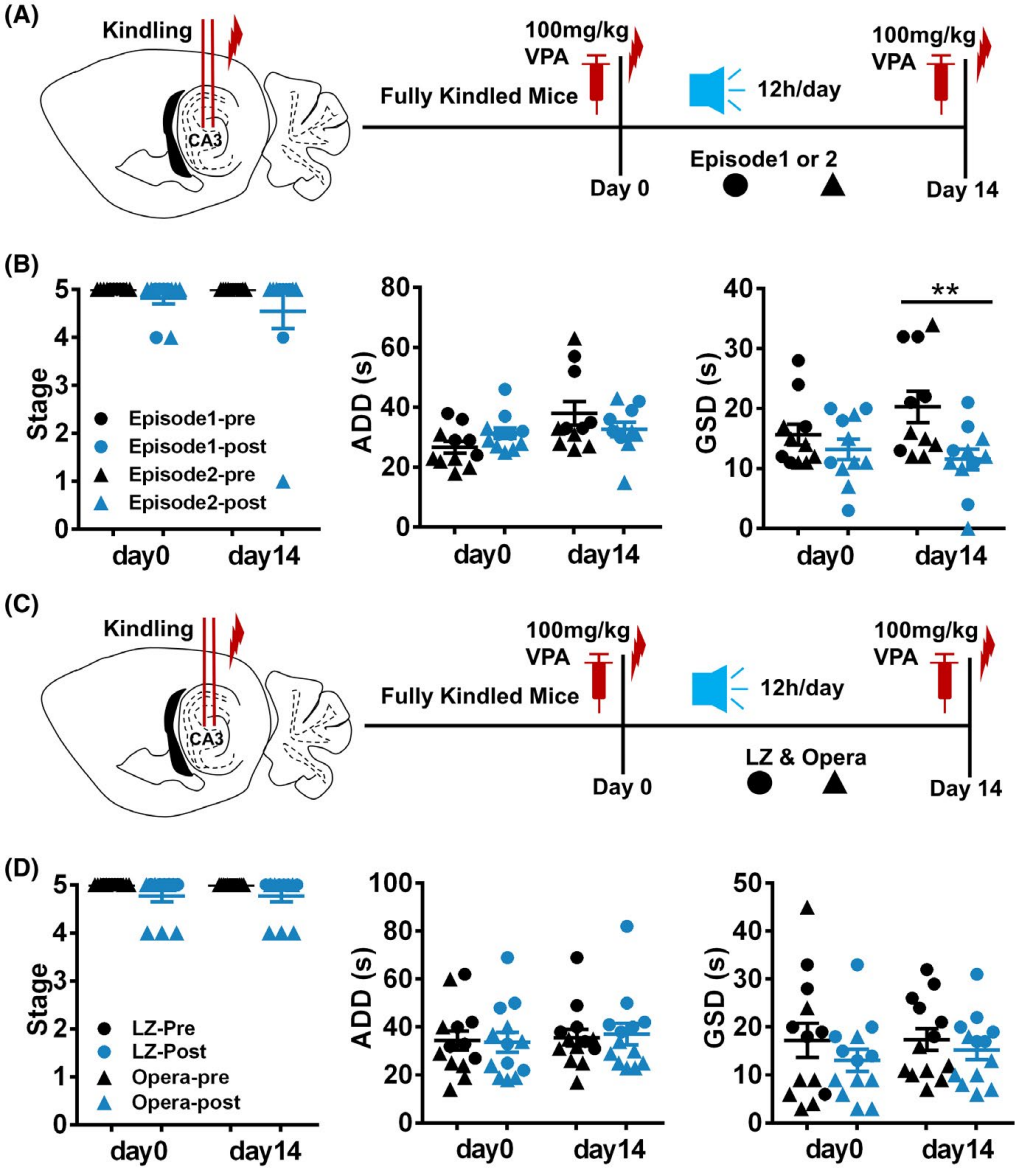
Full‐length Mozart K.448 is necessary for enhancing the anti‐seizure efficacy of VPA on hippocampal kindled mice. (A) The schematic diagram of the experimental design for applying episode 1 or 2, extracted from the full‐length Mozart K.448. (B) Neither episode 1 (n = 6, marked with filled circles) nor episode 2 (n = 5, marked with filled triangles) showed an effect comparable with full‐length Mozart K.448 on hippocampal kindled mice. (C) The schematic diagram of the experiment design for applying LZ and opera, instead of full‐length Mozart K.448. (D) Neither LZ (n = 6, marked with filled circles) nor opera (n = 7, marked with filled triangles) showed any effect on promoting the anti‐seizure efficacy of 100 mg/kg VPA on hippocampal kindled mice

The Mozart effect has been previously attributed to the repetitive rhythm of Mozart K.448.^23^ Given that the whole of Mozart K.488 comprises several distinct parts, we endeavored to uncover which piece of Mozart K.448 plays the decisive role in its therapeutic effects. We divided Mozart K.448 into two parts according to its musicality: episode I (0:00–2:27) is the main repetitive chapter of Mozart K.448, while episode II (6:32–8:24) is the evolving chapter with the most changes in the rhythm. We then tested the effect of these two episodes in hippocampal kindled mice (Figure 5A). After exposing kindled mice to Episode 1 or Episode 2 for 14 days (12 h/day) we then tested the effects of 100 mg/kg VPA. Neither Episode 1 nor Episode 2 alone mimicked the effect of the full‐length Mozart K.448 (Figure 5B), indicating that the full Mozart K.448 is necessary.

We then tested whether different classics or varieties of music could induce a similar effect to Mozart K.448. To answer this question, Chinese traditional music, both the LiangZhu Butterfly Lovers Violin Concerto (LZ) and traditional Peking Opera (opera), were chosen. The protocol of listening to LZ and opera was identical to the Mozart K.448 protocol (Figure 5C). However, neither LZ nor the opera could enhance the anti‐seizure efficacy of sub‐dose VPA on hippocampal kindled mice at day 14 (Figure 5D).

In summary, our results suggest that only 12 h/day full‐length Mozart K.448 is capable of enhancing the anti‐seizure efficacy of AEDs in kindled mice.

### 3.4 Mozart K.448 adjunctive therapy modulates hippocampal seizure EEG spectral power in the RES group

**FIGURE 6:**
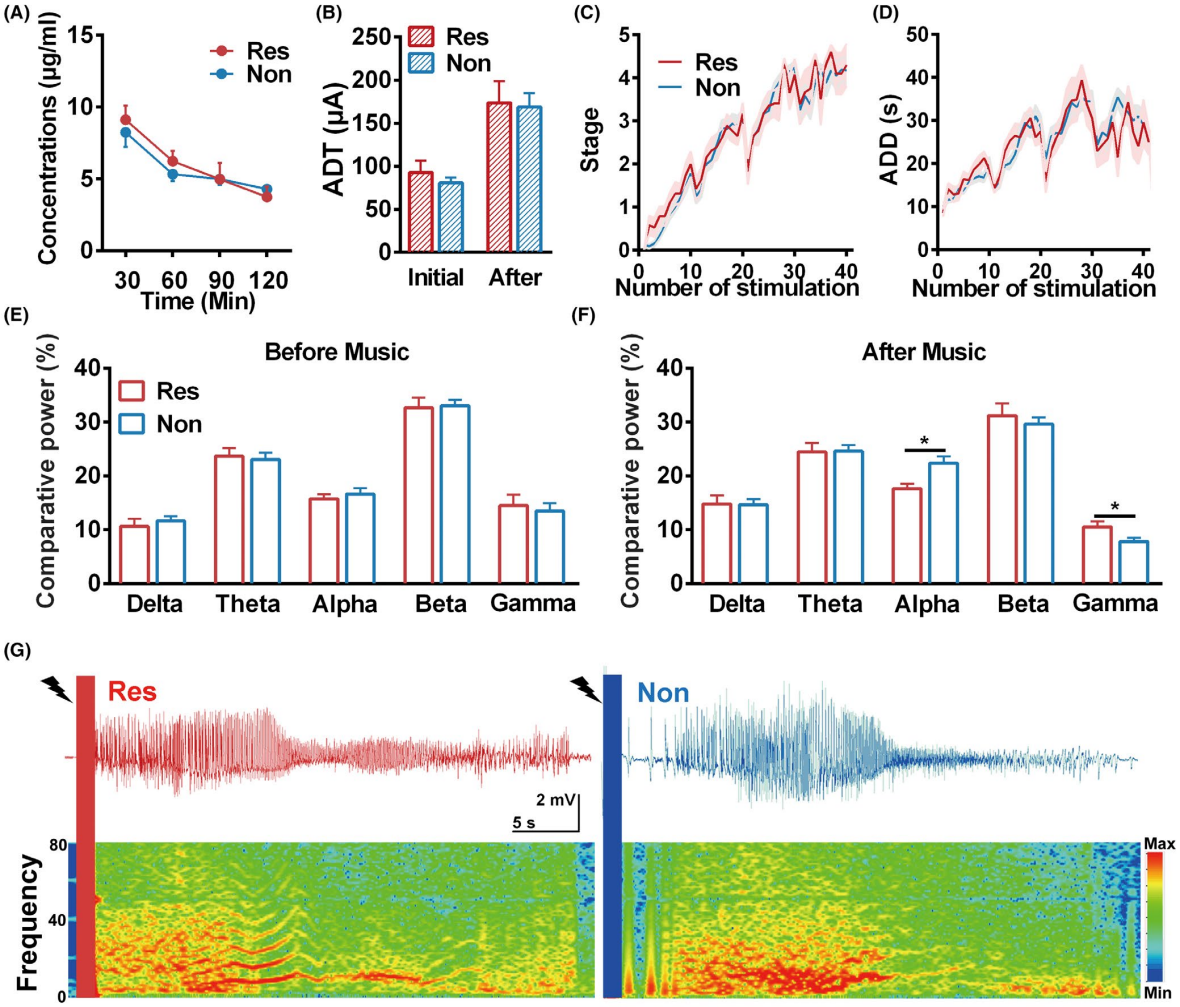
Mozart K.448 modulates seizure EEG spectrum in mice of the RES group. (A) VPA concentration time profiles in right hippocampal dialysate (n = 3 for the RES group, n = 4 for the NON group). No significant differences were found between two groups. (B) The ADTs obtained before kindling and at day 14 showed no difference between the RES and NON groups in hippocampal kindled mice (n = 19 for the RES group, n = 32 for the NON group). (C and D) The development of seizure stage (C) and ADD (D) during the kindling acquisition process showed no differences between the RES and NON groups (n = 19 for the RES group, n = 32 for the NON group). (E) Comparative spectrum power of seizure EEGs in the RES and NON groups before music application. The comparative power of all the analyzed frequency bands showed no differences (n = 17 for the RES group, n = 29 for the NON group). (F) The comparative spectrum power of seizure EEGs in the RES and NON groups after Mozart K.448 music application for 14 days. Seizure EEGs of the RES group showed lower comparative power in the alpha band while higher in the gamma band after music application (n = 17 for the RES group, n = 29 for the NON group, for alpha band, ^*^ *p* = 0.011, unpaired t‐test was used; for gamma band, ^*^ *p* = 0.028, nonparametric Mann‐Whitney test was used). (G) Representative seizure EEGs and power spectrums in mice from the RES and NON groups [Colour figure can be viewed at wileyonlinelibrary.com]

Given that mice experienced listening to Mozart K.448 show a distinct response to sub‐dose AEDs, we further want to discover the deeper differences between the RES and NON groups. As drug efficacy usually depends on its concentration in the brain,^2^ we examined whether or not Mozart K.448 could raise the intracerebral concentration of VPA in the RES group. As shown in Figure 6, the VPA concentration of the hippocampal cerebrospinal fluid showed no difference between the RES and NON groups at each time point (Figure 6A). This result indicates that the effect of Mozart K.448 was not due to the enhanced drug concentration in the brain. Due to that seizure susceptibility also shows impact on the AED’s anticonvulsant effects. We then retrospectively compared the ADTs and kindling acquisition process between these two groups in the hippocampal kindling model. The initial ADTs and the ADTs after listening to music showed no difference between two groups (Figure 6B), which indicates that the music did not influence seizure susceptibility. Similarly, the progression of seizure stage and ADDs also exhibited no difference (Figure 6C‐D), suggesting that there is no difference in basic epileptogenic susceptibility between the two groups.

To further investigate the influence of Mozart K.448 on seizure activity, we analyzed the spectrum power of hippocampal seizure EEGs. The power of all basic bands (0–100 Hz) showed no differences between these two groups before music application (Figure 6E). However, after applying Mozart K.448 for 14 days, the alpha and gamma band power showed significant differences between the RES and NON groups (the relative power of the alpha band was lower while that of the gamma band was higher in the RES group compared with the NON group; Figure 6F). The representative seizure EEG and corresponding power spectrum of RES and NON groups after Mozart K.448 application verified this (Figure 6G). These results indicate that Mozart K.448 may alter the alpha and gamma band power of the hippocampal seizure EEGs in those mice that responded to the treatment.

## 4 DISCUSSION

In the present study, we provide the first *in vivo* evidence that listening to Mozart K.448 for 12 h/day is a potential adjuvant therapy for TLE models by enhancing the anti‐seizure efficacy of AEDs. Although other adjuvant therapies such as alternative medications, neuromodulation, and ketogenic diets have been used previously to facilitate controlling TLE seizures, our findings reveal that Mozart K.448 therapy has its own advantages. First, in modern studies of adjuvant therapies on epilepsy, AEDs are usually administered as normal, with the adjuvant therapies added in an auxiliary capacity. Mozart K.448 therapy was able to not only enhance the anti‐seizure effect of VPA at its therapeutic dosage but also enhance the efficacy of sub‐dose VPA and LEV (Figures 1 and 4). And the magnitudes of anti‐seizure effects (indicated by the reduction ratio of seizure duration) achieved by music adjuvant therapy plus sub‐dose VPA are analogous to that achieved by therapeutic dose VPA on kindled model^31^, ^32^ and intrahippocampal kainate model,^33^ and even prior to other pharmacological compounds.^34^, ^35^ As TLE patients normally need AEDs in a long‐term or even life‐long manner, continuous medication can induce unwanted side effects and economic burdens. The reduced minimum dose threshold of AEDs facilitated by Mozart K.448, an effect rarely observed in other adjuvant therapies, would relieve both physical and economic burdens caused by long‐term medications for TLE patients. Second, epilepsy patients typically show a good response to AEDs at first, but in many patients the drug effect becomes poorer over time.^36^, ^37^ Here, we found that the effect of Mozart K.448 becomes more prominent over time on various TLE models, suggesting that tolerance may not be a concern in music adjuvant therapy. Lastly, both neuromodulation and ketogenic diets need the assistance of professional facilities or clinicians to be successful. Music adjuvant therapy, on the other hand, is convenient and can be self‐administered. Given the advancements in modern electronics, patients could receive candidate music through their smartphones and headsets without invasive risks or interruptions to daily life. However, there still exists some important points that should not be ignored, one is the potential evolutionary differences of musical information procession between rodents and humans, which might restrict the translational significance of this study at a certain degree. The other is the models we used in this study is not typical drug‐resistant TLE model, whether music adjuvant therapy is also effective for drug‐resistant TLE still needs further investigation. However, given the desirable adjuvant therapeutic effect on kindled seizures has been observed, we can optimistically speculate that patients with drug‐resistant TLE may also benefit from music adjuvant therapy. If yes, it would be safer and more convenient to achieve therapeutic effects than current used invasive therapies such as deep brain stimulation and vagal nerve stimulation.^38^, ^39^ In summary, music adjuvant therapy could enhance the anti‐seizure efficacy of sub‐dose AEDs and provide a promising and convenient noninvasive adjuvant therapy for TLE.

The selection of appropriate protocols is a critical issue for music adjuvant therapy: inconsistent findings have been shown across numerous studies with differing protocols.^13^, ^14^, ^15^, ^16^, ^17^, ^40^ Notably, highly variable music durations—ranging from 30 min to 6 h/day of listening—have been used in previous studies.^14^, ^15^, ^16^, ^40^ In this study, we evaluated several durations of Mozart K.448 listening and found only 12 h/day Mozart K.448 to be efficacious, with shorter duration music applications (2 h or 6 h per day) proving ineffective in enhancing the anti‐seizure efficacy of AEDs. Our result suggests that listening to Mozart K.448 for 12 h/day might be an optimal. In addition to listening duration, the music selection is highly important. Although music therapy has caught more and more attention in treating neurological diseases, and candidate music including various types from classic to pop music,^41^ however, it seems that in epilepsy, only Mozart K.448 had a desirable effect rather than other sonatas and symphonies.^40^, ^42^ In this study, we further verified that neither an individual episode of Mozart K.448, nor other types of tested music had an adjuvant therapeutic effect. A possible mechanism by which Mozart K.448 might exert its novel effect could be its special periodicity, indeed, a 10–60 s long‐term periodicity is found most frequently in Mozart K.448 compared with other sonatas created by different composers.^42^ Although music can directly modulate emotion‐related functions as an immemorial art form, the effect of Mozart K.448 is more likely related to its periodicity and the resulting brain resonation, rather than its effect on emotional functions.^23^ Because of these potential mechanisms and our own findings, we put forth listening to full‐length Mozart K.448 12 h/day as a new gold standard for music adjuvant therapy in TLE seizures, against which variations to the listening duration or music selection should be evaluated. Furthermore, we suggest when searching for new musical candidates, researchers should pay special attention to the periodicity.

The potential mechanisms of music adjuvant therapy are complicated and obtuse. In this study, neither innate epileptogenic susceptibility nor intracerebral drug concentration is involved in the therapeutic effect of Mozart K.448 on the RES group. However, Mozart K.448 changed relative spectrum powers of seizure EEGs in responsive mice, indicating that Mozart K.448 might influence hippocampal neural activities. Given that the effect of Mozart K.448 only appeared when applied in a 12 h/day manner, and its effect was more prominent in day14 than day7 (Figures 1 and 2), we can propose that Mozart K.448 probably has a long‐term depotentiation effect on the responsive mice. Further EEG analysis showed that Gamma band power which is previously reported to be related with inhibitory GABAergic neural activities^43^ was increased in the RES group after listening to Mozart K.448. Based on this, it is highly possible that long‐term Mozart K.448 induced long‐term synaptic plasticity changes in auditory regions and then increased the hippocampal GABAergic neural activities through auditory circuits.^44^, ^45^ Interestingly, long‐term Mozart K.448 did not achieve a 100% effective rate in mice (~42% mice were responsive in this study). It is likely that a diversity of neural circuitry responses to Mozart K.448 exists in mice, just like in humans where differences in the neural response to music have been reported.^46^ And music sensitivity should be examined where possible before arranging individual patients to receive music adjuvant therapy.

To conclude, we found that Mozart K.448 adjuvant therapy can enhance the anti‐seizure efficacy of different sub‐dose AEDs in two kindled TLE models. Furthermore, we identify a gold standard template for reviewers to compare alternative music therapy strategies and determine their relative efficacy. Our promising preclinical data shed a new light on applying Mozart K.448 as an effective adjuvant therapy in TLE.

## CONFLICTS OF INTEREST

No conflicts to report.

## Data Availability

The authors confirm that the data supporting the findings of this study are available within this article and from the corresponding author upon reasonable request.
